# Homeostatic regulation of NAD(H) and NADP(H) in cells

**DOI:** 10.1016/j.gendis.2023.101146

**Published:** 2023-10-17

**Authors:** Luojun Chen, Xiaoke Xing, Pingfeng Zhang, Lulu Chen, Huadong Pei

**Affiliations:** a Cancer Center, Renmin Hospital of Wuhan University, Wuhan, Hubei 430062, China; b Department of Oncology, Georgetown Lombardi Comprehensive Cancer Center, Georgetown University Medical Center, Washington, DC 20057, USA

**Keywords:** MESH1, NAD(H), NADK, NADP(H), Nocturnin

## Abstract

Nicotinamide adenine dinucleotide (NAD^+^)/reduced NAD^+^ (NADH) and nicotinamide adenine dinucleotide phosphate (NADP^+^)/reduced NADP^+^ (NADPH) are essential metabolites involved in multiple metabolic pathways and cellular processes. NAD^+^ and NADH redox couple plays a vital role in catabolic redox reactions, while NADPH is crucial for cellular anabolism and antioxidant responses. Maintaining NAD(H) and NADP(H) homeostasis is crucial for normal physiological activity and is tightly regulated through various mechanisms, such as biosynthesis, consumption, recycling, and conversion between NAD(H) and NADP(H). The conversions between NAD(H) and NADP(H) are controlled by NAD kinases (NADKs) and NADP(H) phosphatases [specifically, metazoan SpoT homolog-1 (MESH1) and nocturnin (NOCT)]. NADKs facilitate the synthesis of NADP^+^ from NAD^+^, while MESH1 and NOCT convert NADP(H) into NAD(H). In this review, we summarize the physiological roles of NAD(H) and NADP(H) and discuss the regulatory mechanisms governing NAD(H) and NADP(H) homeostasis in three key aspects: the transcriptional and posttranslational regulation of NADKs, the role of MESH1 and NOCT in maintaining NAD(H) and NADP(H) homeostasis, and the influence of the circadian clock on NAD(H) and NADP(H) homeostasis. In conclusion, NADKs, MESH1, and NOCT are integral to various cellular processes, regulating NAD(H) and NADP(H) homeostasis. Dysregulation of these enzymes results in various human diseases, such as cancers and metabolic disorders. Hence, strategies aiming to restore NAD(H) and NADP(H) homeostasis hold promise as novel therapeutic approaches for these diseases.

## Introduction

Nicotinamide adenine dinucleotide (NAD^+^)/reduced NAD^+^ (NADH) and nicotinamide adenine dinucleotide phosphate (NADP^+^)/reduced NADP^+^ (NADPH) are indispensable molecules in various metabolic processes, such as biosynthesis, consumption, recycling, and conversion in cells.^1^^,^^2^ Additionally, NAD(H) and NADP(H) are directly implicated in an array of cellular activities, including mitochondrial energy generation, redox metabolism and homeostasis, signal transduction, genomic stability, gene expression regulation, antioxidation, circadian clock management, immunity, and inflammation.^1^^,^^2^ Dysregulation of NAD(H) and NADP(H) homeostasis can trigger numerous pathological changes, ultimately leading to various human diseases.

### Physiological roles of NAD(H)

NAD^+^ and NADH form a critical redox couple, central to controlling energy metabolism.^3^ NAD^+^ gains a charged hydrogen molecule (H^+^) and two electrons to convert to NADH, while NADH, serving as a donor of H^+^ and electrons, is converted back to NAD^+^.^4^ This NAD^+^-NADH cycling is essential for the continuous flow of H^+^ and electrons in the cytosol and mitochondria, contributing to adenosine triphosphate (ATP) generation through the electron transport chain.^4^

NAD^+^ also functions as an essential co-substrate for non-redox NAD^+^-dependent enzymes (also referred to as NAD^+^-consuming enzymes), including sirtuins (SIRTs), poly (adenosine diphosphate-ribose) polymerases (PARPs), CD38, CD157, and sterile alpha and toll/interleukin-1 receptor motif-containing 1 (SARM1).^1^ These enzymes cleave NAD^+^ to produce nicotinamide, ADP-ribosylation (ADPR), or cyclic ADP ribose (cADPR), crucial for various post-synthetic modifications of essential macromolecules.^5^ For instance, SIRTs, found in different cellular compartments, cleave acetyl groups from lysine residues of histones or other proteins and break down NAD^+^ to produce ADPR and nicotinamide.^6^ PARPs use NAD^+^ as a co-substrate to add monomer or polymer ADPRs to target proteins,^7^, ^8^, ^9^ crucial for DNA damage response and genome stability.^10^, ^11^, ^12^ Additionally, cADPR synthetases (CD38, CD157) cleave NAD^+^ to release cADPR, a critical calcium-mobilizing second messenger that modulates key cellular processes such as metabolism and immune cell activation.^13^, ^14^, ^15^, ^16^, ^17^

### Physiological roles of NADP(H)

**Figure 1:**
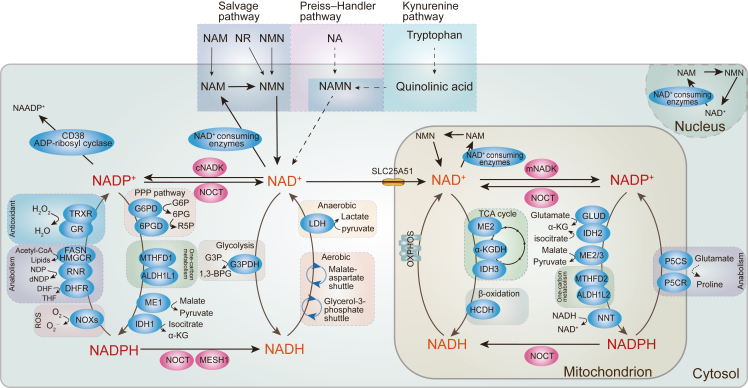
NAD^+^ synthesis and conversions between NAD^+^, NADH, NADP^+^, and NADPH. NAD^+^ can be synthesized from tryptophan via the kynurenine pathway or from nicotinic acid (NA) via the Preiss-Handler pathway. It can also be generated from nicotinamide (NAM), nicotinamide riboside (NR), and nicotinamide mononucleotide (NMN) through the salvage pathway. NAD^+^ is consumed by NAD-consuming enzymes (such as SIRTs, PARPs, CD38, CD157, and SARM1), producing NAM as a byproduct. NAD^+^ is reduced to NADH by receiving H^+^ from glycolysis, fatty acid oxidation (FAO), and the tricarboxylic acid (TCA) cycle. NADH is then oxidized back to NAD^+^ by donating H^+^ during oxidative phosphorylation (OXPHOS), generating ATP and converting pyruvate to lactic acid. Furthermore, NADH is exchanged between the cytosol and mitochondria through the malate-aspartate shuttle and glycerol-3-phosphate shuttle, supporting OXPHOS in mitochondria. NAD^+^ can be phosphorylated directly by NADK to generate NADP^+^. Subsequently, NADP^+^ is reduced to NADPH by various enzymes like glucose-6-phosphate dehydrogenase (G6PD), 6-phosphogluconate dehydrogenase (6PGD), and malic enzyme 1 (ME1) in the cytoplasm, by isocitrate dehydrogenase 2 (IDH2), glutamate dehydrogenase (GLUD), nicotinamide nucleotide transhydrogenase (NNT), and malic enzyme 3 (ME3) in mitochondria, and by cytosolic methylenetetrahydrofolate dehydrogenase 1 (MTHFD1)/aldehyde dehydrogenase 1 family member L1 (ALDH1L1) and mitochondrial methylenetetrahydrofolate dehydrogenase 2 (MTHFD2)/aldehyde dehydrogenase 1 family member L2 (ALDH1L2) in folate-mediated one-carbon metabolism. Conversely, NADPH is oxidized to NADP^+^ by providing reducing equivalents during antioxidant processes and biosynthesis. Enzymes like glutathione reductases (GR) and thioredoxin reductases (TRXR) use NADPH to maintain the reduced form of glutathione (GSH) and TRX-(SH)_2_, respectively, reducing H_2_O_2_ or other peroxides to H_2_O. The enzyme cytochrome P450 reductases use NADPH as a cofactor in drug and xenobiotic metabolism. NADPH also provides reducing equivalents for fatty acid synthesis through FASN, for cholesterol and nonsterol isoprenoids synthesis through HMGCR, and for proline synthesis through pyroline-5-carboxylate synthase (P5CS) and pyroline-5-carboxylate reductases (P5CR) in mitochondria. Dihydrofolate reductase (DHFR) also utilizes NADPH in folate metabolism to catalyze the reduction of dihydrofolate to tetrahydrofolate (THF). Ribonucleotide reductase (RNR) uses NADPH to catalyze the reduction of ribonucleotide 5′-diphosphate (NDP) to deoxyribonucleotide diphosphate (dNDP). Additionally, NADP(H) can be converted back into NAD(H) under the catalysis of NADP(H) phosphatases like NOCT and MESH1. Furthermore, NADP^+^ can be converted to nicotinic acid adenine dinucleotide phosphate (NAADP^+^) by CD38 and ADP-ribosyl cyclase, triggering Ca^2+^ release.

NADP^+^ and NADPH constitute another critical redox pair, influencing a wide array of biochemical reactions. While NADP^+^ functions as a coenzyme for NADP^+^-dependent dehydrogenation reactions, NADPH acts as a donor of H^+^ and electrons, participating in antioxidative stress responses and various anabolic reactions.^2^ It provides the necessary reducing equivalents for the synthesis of antioxidant molecules and essential biological macromolecules, such as fatty acids (catalyzed by fatty acid synthase (FASN)), steroids (cholesterol and nonsterol isoprenoid synthesis catalyzed by 3-hydroxy-3-methylglutaryl-CoA reductase (HMGCR)), amino acids, and nucleotides.^2^^,^^18^^,^^19^ NADPH is utilized by enzymes like glutathione reductases and thioredoxin reductases to maintain the reduction of antioxidant molecules like glutathione and TRX-(SH)_2_, reducing harmful hydrogen peroxide or other peroxides to harmless H_2_O.^20^^,^^21^ It also supports drug and xenobiotic metabolism through cytochrome P450 reductases.^22^ In addition, mitochondrial NADPH offers reducing equivalents for proline synthesis, a critical step in the *de novo* biosynthesis of certain amino acids. This process is catalyzed by pyroline-5-carboxylate synthase (P5CS) and pyroline-5-carboxylate reductases (P5CR).^23^^,^^24^ Furthermore, NADPH is also a crucial factor in folate metabolism, as it is utilized by dihydrofolate reductase to catalyze the reduction of dihydrofolate to tetrahydrofolate (THF). This pathway is indispensable for the *de novo* biosynthesis of thymidylate, purine, methionine, and certain essential amino acids.^25^ Additionally, ribonucleotide reductase consumes NADPH during DNA replication, catalyzing the reduction of ribonucleotide 5′-diphosphate to deoxyribonucleotide diphosphate, which is further converted to deoxyribonucleotide triphosphate to fuel DNA replication (Fig. 1).^2^^,^^26^

Beyond supplying reducing equivalents, NADPH also contributes to the generation of reactive oxygen species (ROS).^27^ NADPH oxidases transfer two electrons from cytosolic NADPH to extracellular oxygen to produce superoxide anion radicals, which are crucial for neutrophils antimicrobial defense during the respiratory burst (Fig. 1).^27^, ^28^, ^29^

In summary, maintaining a balanced regulation of NAD(H) and NADP(H) homeostasis is essential for normal cellular functioning. Any disruptions in this balance can lead to various pathological conditions and human diseases.

## NAD^+^ synthesis and conversions between NAD^+^, NADH, NADP^+^, and NADPH

### NAD^+^ synthesis

NAD^+^ is a pivotal molecule involved in the biosynthesis of NADH, NADP^+^, and NADPH. In mammalian cells, three distinct pathways contribute to NAD^+^ synthesis. First, NAD^+^ can be synthesized *de novo* from dietary tryptophan via the kynurenine pathway.^30^ Second, NAD^+^ can be produced from the vitamin B_3_ family through the Preiss-Handler pathway.^1^ The third pathway, known as the salvage pathway, contributes to the majority of cellular NAD^+^. This pathway recycles nicotinamide, a byproduct generated by NAD^+^-consuming enzymes such as SIRTs, PARPs, CD38, CD157, and SARM1. Furthermore, it recycles nicotinamide riboside, nicotinamide, and nicotinamide mononucleotide absorbed from dietary sources (Fig. 1).^31^^,^^32^

### Conversions between NAD^+^ and NADH

The interconversion between NAD^+^ and NADH primarily occurs via the action of NAD^+^-dependent dehydrogenases during cellular metabolism, such as glycolysis, the tricarboxylic acid cycle (also known as the Krebs cycle), and fatty acid oxidation (FAO). For instance, in glycolysis, the oxidation of glyceraldehyde-3-phosphate to 1,3-bisphosphoglycerate by glyceraldehyde-3-phosphate dehydrogenase accompanies the reduction of cytosolic NAD^+^ to NADH.^33^ Similarly, within the tricarboxylic acid cycle, the oxidative decarboxylation of isocitrate by isocitrate dehydrogenase, α-ketoglutarate by α-ketoglutarate dehydrogenase, and malate by malic enzyme coincides with the reduction of mitochondrial NAD^+^ to NADH.^34^, ^35^, ^36^ In FAO, hydroxyacyl-CoA dehydrogenase catalyzes the oxidation of straight-chain 3-hydroxyacyl-CoAs, coupled with the conversion of mitochondrial NAD^+^ to NADH.^37^

In reverse, NADH can be converted back into NAD^+^ via NADH-dependent oxidases, such as lactate dehydrogenase and respiratory chain complexes. Under aerobic conditions, NADH generated in mitochondria can directly transfer electrons to the electron transport chain.^4^ However, cytosolic NADH is unable to cross the mitochondrial inner membrane. Instead, it utilizes the malate-aspartate shuttle or glycerol-3-phosphate shuttle, to transfer electrons to the electron transport chain, leading to the regeneration of NAD^+^ from NADH.^38^^,^^39^ Under anaerobic conditions, when mitochondrial respiration is compromised, the cytosolic reduction of pyruvate to lactate pairs with the regeneration of NAD^+^ from NADH, facilitating glycolysis (Fig. 1).^4^^,^^40^

### Conversions between NADP^+^ and NADPH

NADPH, acting as a crucial donor of H^+^ and electrons, plays a vital role in antioxidative stress responses and anabolic reactions.^2^ It can be produced from NADP^+^ by various dehydrogenases, including glucose-6-phosphate dehydrogenase (G6PD) and 6-phosphogluconate dehydrogenase, which are involved in the pentose phosphate pathway^41^; NADP^+^-dependent malic enzymes (malic enzyme 1 in the cytosol and malic enzyme 2/3 in the mitochondria), which catalyze the transformation of malate to pyruvate, producing NADPH^42^^,^^43^; and NADP^+^-dependent isocitrate dehydrogenase (isocitrate dehydrogenase 1 in the cytosol and isocitrate dehydrogenase 2/3 in the mitochondria), which catalyzes the conversion of isocitrate to α-ketoglutarate,^44^ accompanied by the generation of NADPH. Additionally, NADP^+^-dependent glutamate dehydrogenase facilitates the transamination of glutamate into α-ketoglutarate, leading to NADPH formation.^45^ Among these metabolic activities, the pentose phosphate pathway contributes to the largest portion of cytoplasmic NADPH production,^46^^,^^47^ accounting for approximately 10%–20% of glucose consumption.^48^^,^^49^ Furthermore, within folate-mediated one-carbon metabolism, methylenetetrahydrofolate dehydrogenase 1 in the cytosol and methylenetetrahydrofolate dehydrogenase 2 in the mitochondria catalyze the oxidation of 5,10-methylene-THF to form 10-formyl-THF. Similarly, 10-formyl-THF dehydrogenases (aldehyde dehydrogenase 1 family member L1 in the cytosol and aldehyde dehydrogenase 1 family member L2 in the mitochondria) catalyze the oxidation of 10-formyl-THF. These reactions coincide with the reduction of NADP^+^ to NADPH.^41^^,^^50^^,^^51^ Additionally, NADP^+^ can also be converted to NADPH through nicotinamide nucleotide transhydrogenase, a protein embedded in the mitochondrial inner membrane that accepts electrons from NADH. Nicotinamide nucleotide transhydrogenase plays a significant role in maintaining NADPH levels, contributing to almost 45 % of total NADPH production in the mitochondrial pool (Fig. 1).^52^

### Conversions between NAD(H) and NADP(H)

NAD^+^ can be directly phosphorylated by NAD kinases (NADKs), enzymes that transfer a phosphate group from ATP to the 2′ position of NAD^+^, thus generating NADP^+^.^53^ Approximately 10% of cellular NAD^+^ is converted into NADP^+^.^54^ Conversely, NADP(H) can be converted back into NAD(H) by NADP(H) phosphatases, such as nocturnin (NOCT) and metazoan SpoT homolog-1 (MESH1).^55^, ^56^, ^57^, ^58^ This conversion allows the cells to adjust their metabolic requirements accordingly (Fig. 1).

In conclusion, NAD(H) and NADP(H) play distinct roles in cellular metabolic processes, with NAD(H) participating in catabolism and NADP(H) contributing to cellular anabolism.^2^^,^^33^ It is noteworthy that higher organisms exhibit spatiotemporal redox heterogeneity, a characteristic prominently displayed by NAD(H) and NADPH.^56^^,^^59^, ^60^, ^61^, ^62^, ^63^, ^64^ The conversions between NAD(H) and NADP(H), regulated by NADKs, MESH1, and NOCT, are crucial for various physiological and pathological cellular activities. Dysregulation of these enzymes is associated with various human diseases.^23^^,^^57^^,^^58^^,^^65^ In the following sections, we will explore recent advances in the research on the regulatory mechanisms of these enzymes.

## NAD kinase, a key enzyme for NADP^+^ synthesis from NAD^+^

### Molecular properties of NAD kinase

NADKs belong to a newly identified superfamily of kinases, along with 6-phosphofructokinases, diacylglyceride kinases, and sphingosine kinases. These kinases are distinguished by a conserved GGDG motif. Within the NADK family, there are two distinct orthologs: cytosolic NAD kinase (cNADK, also known as NADK or NADK1) and mitochondrial NAD kinase (mNADK, also recognized as NADK2).^66^, ^67^, ^68^ Both cNADK and mNADK share essential features: a GGDG motif, an NE/D short motif, and a glycine-rich conserved domain II, all crucial for substrate binding and activation.^66^ However, mNADK contains an exclusive N-terminal mitochondrial targeting sequence, which directs its localization to the mitochondria.^67^

Despite these commonalities, the two NADKs show striking differences in higher-order structure. cNADK forms a tetramer composed of two identical dimers.^66^^,^^68^ In contrast, mNADK forms a dimer with two-fold symmetry, mediated by a unique helical extension at amino acids 325–365.^69^ This helical extension (encoding alpha-helices α8 and α9), known as elements conserved in mitochondrial kinases of animals (EMKA), is present in mNADK from drosophila to humans but absent in cNADK.^69^^,^^70^ Mechanistically, EMKA prevents the side-by-side dimerization of mNADK dimers into a tetrameric structure. This is achieved by generating a long hairpin formed of two helical segments (327–340 and 350–364) that interact with their equivalent counterparts from the second aptamer.^69^^,^^70^ This structural distinction between the cNADK and mNADK results in different activation patterns. Tetrameric cNADK is cooperatively activated through oligomerization, while mNADK is constitutively active, ensuring its functionality even in mitochondria-deficient conditions.^69^^,^^70^

### Physiological function of NAD kinases

NADKs, including cNADK and mNADK, serve as the key enzymes responsible for the *de novo* synthesis of NADP^+^ in mammalian cells. They transfer the phosphate group from ATP to the 2′ position of the ribose ring attached to the adenine part in NAD^+^, producing NADP^+^.^53^ While studies suggest that mNADK can phosphorylate both NAD^+^ and NADH using either ATP or polyphosphate as the phosphate source (as observed in bacteria and archaea), it predominantly prefers NAD^+^ and ATP as substrates in mammalian cells.^66^^,^^67^

NADKs play pivotal roles in regulating energy metabolism, antioxidative stress, proline synthesis, and cell growth.^24^^,^^71^^,^^72^ For instance, in *Saccharomyces cerevisiae*, the combined knockout of cytosolic NAD kinase-UTR1 and mitochondrial NAD kinase-POS5 resulted in synthetic lethality.^73^ In mice, systemic cNADK knockout led to either embryonic or preweaning lethality.^74^ In pancreatic β-cells, cNADK plays an indispensable role in glucose-stimulated insulin secretion, as NADPH acts as a coupling mediator.^75^ In fact, studies have shown a 30% increase in glucose-stimulated insulin secretion upon overexpression of cNADK, and cNADK knockdown induces a significant inhibition of the secretion in a pancreatic insulinoma cell line.^76^ Moreover, in *Drosophila*, the depletion of NADK impairs lipid storage and mitochondrial functions,^65^^,^^77^ highlighting its significance in metabolic regulation.

mNADK has garnered significant interest due to its role in protecting cells from harmful oxidative stresses and regulating FAO by modulating mitochondrial NADPH biosynthesis.^71^ Defective mNADK results in reduced NADPH levels, leading to a significant accumulation of ROS.^4^^,^^71^ Increased ROS levels can partly influence the activity of SIRTs and alter the transcription of downstream genes, such as cyclic adenosine monophosphate responsive element-binding protein H (CREBH), peroxisome proliferator-activated receptor-α (PPARα), and peroxisome proliferator-activated receptor gamma coactivator 1-α.^72^ CREBH and PPARα can regulate mitochondrial FAO, which is crucial for energy generation during starvation.^72^^,^^78^ In summary, a decrease in mNADK activity can lead to elevated ROS levels and FAO dysfunction, contributing to insulin tolerance and hepatic steatosis.^71^^,^^72^^,^^79^

Some studies suggest that mNADK deficiency does not affect the folate metabolic pathway or increase oxidative stress,^23^^,^^24^ revealing a potential area for further exploration. Two recent studies have shed light on the significant role of mNADK in promoting proline biosynthesis, essential for synthesizing nucleotides and proteins during growth.^23^^,^^24^ In the mitochondria, P5CS converts glutamine-derived glutamate to pyrroline-5-carboxylate (P5C), and P5CR further reduces P5C to proline. The production of P5C through P5CS is a bottleneck stage and relies heavily on mNADK activity and NADPH level.^80^ mNADK-deficient cells become proline auxotrophic, indicating its critical role in proline production.^23^^,^^24^ Genetic deficiencies in mNADK have severe consequences. Homozygous mNADK knockout in mice results in embryonic lethality, while severe genetic mNADK deficiency in humans leads to neurological and developmental impairments, causing conditions such as encephalopathy, microcephaly, epilepsy, and early death between four months and five years after birth.^81^, ^82^, ^83^

## Regulation of cNADK

### Transcriptional regulation of cNADK

cNADK is subject to transcriptional regulation, as well as degradation mediated by miR-690 in M2-polarized bone marrow-derived macrophages (BMDMs). This process aids in the maintenance of macrophage repolarization from an M1 to an M2 state. Up-regulation of miR-690 in BMDMs leads to a significant decrease in cNADK expression, promoting anti-inflammatory effects.^84^ Notably, miR-690 is abundant in exosomes released by M2-polarized BMDMs, which can transport it to the liver and adipose tissue. In these tissues, miR690 inhibits cNADK, resulting in improved glucose intolerance and insulin resistance.^84^ Treatment with exosomes from M2-polarized BMDMs has been shown to stimulate glucose uptake and alleviate obesity-associated inflammation in adipose tissue of obese mice fed a high-fat diet.^84^ Kupffer cells, which are hepatic macrophages, also exhibit high levels of endogenous miR-690.^85^ This miR-690 enhances Kupffer cell self-proliferation by inhibiting cNADK.^86^ Moreover, Kupffer cells can also shuttle miR-690 to neighboring hepatic cells or hepatic stellate cells through secreted exosomes, leading to cNADK inhibition in these cells. The inhibition of cNADK mediated by miR-690 can alleviate fibrosis and inflammation in models of nonalcoholic steatohepatitis (NASH). Mice with Kupffer cell-specific miR-690 knockout showed enhanced fibrosis and other NASH characteristics.^86^

**Figure 2:**
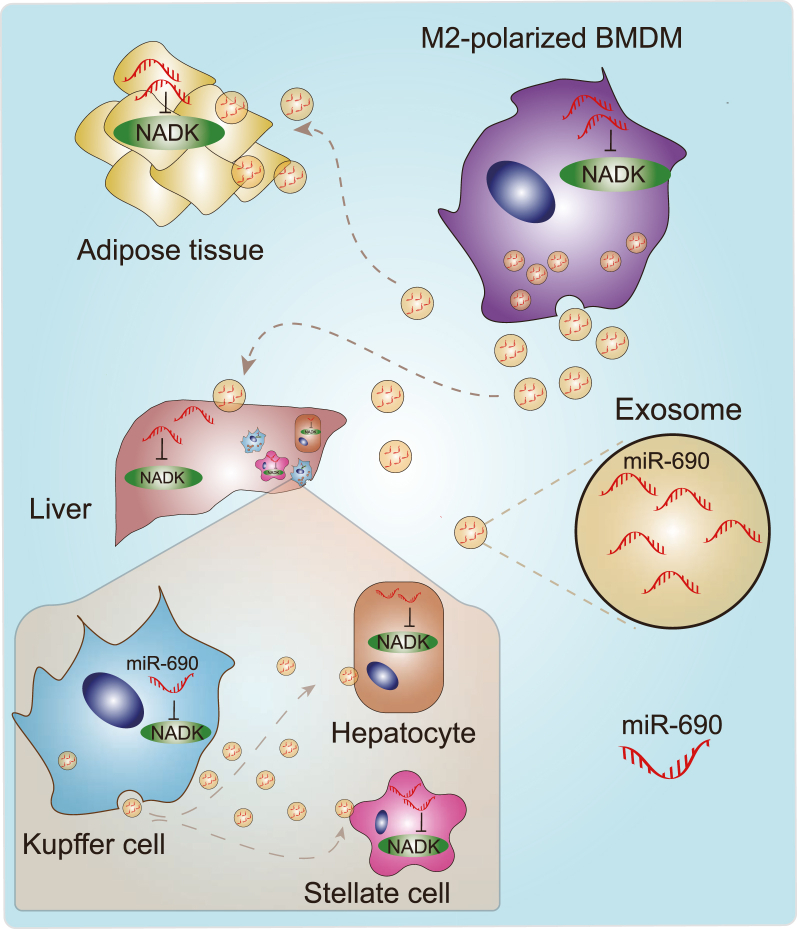
miR-690 inhibits cytosolic NAD kinase (cNADK). In M2-polarized bone marrow-derived macrophages (BMDMs), miR-690 targets cNADK mRNA for degradation. Additionally, miR-690 can be transported via exosomes to the liver and adipose tissue, where it inhibits cNADK, leading to improved glucose tolerance and insulin resistance. Kupffer cells, characterized by high levels of endogenous miR-690, employ it to inhibit cNADK, promoting their self-proliferation. Furthermore, Kupffer cells can transmit miR-690 to neighboring hepatic cells or hepatic stellate cells via exosome secretion. This leads to cNADK inhibition in these cells, thereby mitigating fibrosis and inflammation in nonalcoholic steatohepatitis models.

While the role of cNADK and its regulation by miR690 in obesity-associated glucose intolerance, insulin resistance, fibrosis, and inflammation are evident, the precise molecular mechanisms involved in these processes require further investigation (Fig. 2). Understanding these intricate regulatory mechanisms may offer potential therapeutic targets for obesity-related metabolic disorders and liver diseases.

### Posttranslational regulation of cNADK

The N-terminal region of cNADK, which is relatively poorly conserved throughout deuterostome evolution, functions as a major activity regulator through posttranslational modifications (PTMs) or protein interactions.^85^ Removal of the N-terminal region (amino acids 1–87) leads to enhanced enzyme activity, suggesting that it functions as an autoinhibitory domain in the native protein.^65^ Notably, PTMs of cNADK predominantly occur in this N-terminal region, implying the existence of regulatory pathways that modulate cNADK enzyme activity by relieving or enhancing its self-inhibitory conformation.

One significant regulator of cNADK is the AKT protein kinase, which phosphorylates cNADK at serine 44, 46, and 48 in the N-terminal region. This phosphorylation releases the self-inhibitory structure, leading to increased cNADK enzyme activity and enhance NADP^+^ production.^65^^,^^87^ Although wild-type and phospho-mutant cNADK exhibit comparable basal enzymatic activity, *in vitro* phosphorylation by AKT only escalates the catalytic activity of wild-type cNADK. Conversely, a cNADK mutant lacking the N-terminal region exhibits constitutively increased basal activity.^65^ These findings propose that the AKT-NADK axis directly stimulates NADP^+^ production through phosphorylation, relieving the N-terminal autoinhibition.^65^

**Figure 3:**
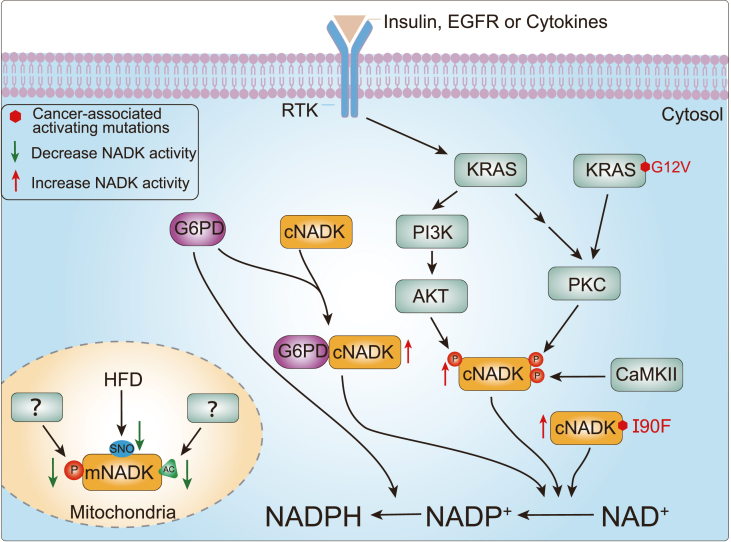
Regulation of NADK activity at the protein level. The growth factor-stimulated phosphoinositide 3-kinase (PI3K)-Akt signaling induces phosphorylation of cytosolic NAD kinase (cNADK) by AKT at serine 44, 46, and 48. Additionally, activated KRAS signaling, such as the gain-of-function mutant KRAS G12V, can phosphorylate cNADK at serine 46 and 64 by PKC. Phosphorylation at serine 44, 46, and 48 promotes the release of cNADK's N-terminal autoinhibitory conformation, enhancing its enzyme activity and boosting the production of NADP^+^. Calmodulin-dependent protein kinase II (CaMKII) increases cNADK enzyme activity by phosphorylating it at serine 64. Glucose-6-phosphate dehydrogenase (G6PD) stimulates cNADK activity by binding to its N-terminus. The gain-of-function mutant (I90F) augments cNADK enzymatic activity by broadening the dimer interface of cNADK, stabilizing the tetrameric organization. Conversely, phosphorylation at serine 188, along with acetylation at lysine 76 and 304, inhibits mNADK activity by blocking substrate binding. Overnutrition, as seen in a high-fat diet (HFD), represses mNADK activity by S-nitrosylating (SNO) mNADK at cysteine 193.

In the context of cellular anabolism, the growth factor-stimulated phosphoinositide 3-kinase (PI3K)-Akt signaling network advances cellular anabolism by either directly phosphorylating key metabolic enzymes or through transcriptional regulation.^88^ These key anabolic processes regulated by this network include the biosynthesis of nucleotides, lipids, and amino acids, which require abundant reducing equivalents in the form of NADPH.^88^ To meet this demand, AKT directly phosphorylates cNADK, enhancing its enzyme activity and stimulating NADP(H) production. This signaling axis provides a precise mechanism to modulate the availability of reducing power in response to growth factor stimulation (Fig. 3).

Protein kinase C (PKC) also contributes to the phosphorylation of cNADK at serine 46 and 64.^89^^,^^90^ Activated KRAS, a well-known oncogenic driver for various tumors, such as pancreatic ductal adenocarcinoma (PDAC) and colon adenocarcinoma (COAD),^91^^,^^92^ enhances cNADK enzyme activity via PKC, boosting NADP^+^ generation and promoting PDAC progression.^90^ Additionally, PKC-mediated cNADK phosphorylation and subsequent NADP^+^ production are essential for the neutrophil respiratory burst response, enhancing the resistance to invading microorganisms (Fig. 3).^89^

A robust correlation exists between the product of NADP^+^, nicotinic acid adenine dinucleotide phosphate, and Ca^2+^ signaling.^15^ The activation of calmodulin-dependent protein kinase II enhances NADP^+^ production by phosphorylating cNADK at serine 64.^85^ Studies propose that glucose-stimulated Ca^2+^ signaling may regulate cNADK activity to augment insulin secretion in pancreatic insulinoma cell lines, thus influencing glucose-stimulated insulin secretion (Fig. 3).^76^ This suggests a potential link between Ca^2+^ signaling, NADP^+^ metabolism, and insulin secretion in response to glucose levels.

### G6PD stimulates cNADK

cNADK undergoes stimulation through its interaction with G6PD at the N-terminus, resulting in increased activity and promoting the production of NADP^+^.^93^^,^^94^ This elevation in NADP^+^ facilitates the conversion to NADPH. Interestingly, elevated expression of G6PD contributes to anchorage-independent growth of immortalized human cells *in vitro* and is associated with tumorigenicity in animals (Fig. 3).^93^^,^^94^ These findings suggest a potential role of G6PD-cNADK interaction in cellular processes related to cell growth and tumorigenesis.

### cNADK mutation

An intriguing gain-of-function screening study identified a low-frequency mutant, known as I90F, which is implicated in promoting tumorigenesis in PDAC.^95^ Structural analysis of cNADK suggests that the I90F mutation, located at the dimer-dimer interface of cNADK, may broaden the cNADK dimer interface. Consequently, this leads to an increase in cNADK enzymatic activity and NADP synthesis, promoting reductive biosynthesis and reinforcing antioxidative stress defenses in PDAC (Fig. 3).^95^

## Regulation of mNADK

### Transcriptional regulation of mNADK

In mice, the expression of mNADK mRNA is influenced by nutritional status. Fasting leads to an elevation of mNADK mRNA levels in the liver and adipose tissue, while a high-fat diet intake results in the opposite effect.^96^

### Posttranslational modifications of mNADK

The regulatory roles of PTMs in mNADK have been elucidated through biochemical and structural analysis. Evidence suggests that negatively charged PTMs (such as phosphorylation and acetylation) can modify local conformations at active sites or neutralize the positive charge on the lysine side chain, thereby influencing the binding of negatively charged molecules such as ATP, NAD^+^, and NADP^+^. These modifications subsequently affect mNADK enzyme activity.^69^ For example, acetylation at lysine 304 hinders NAD^+^ from binding to mNADK, and acetylation at lysine 76 interferes with ATP binding.^69^ Moreover, phosphorylation at serine 188 prevents the phosphate group of ATP from transferring to NAD^+^. Replacing serine with aspartate, glutamate, or even alanine at position 188 of mNADK suppresses its activity.^97^ In conclusion, both phosphorylation and acetylation are crucial in regulating mNADK catalytic efficiency, and play an essential role in mitochondrial NADP(H) production, proline synthesis, and cell proliferation (Fig. 3).

S-nitrosylation, similar to phosphorylation and acetylation, modulates numerous functions of mNADK by altering its enzymatic activity.^71^ Nutrition overload inhibits mNADK activity through S-nitrosylation at cysteine 193, leading to decreased mitochondrial NADP(H) levels and increased cellular ROS levels.^71^ This inhibition also results in insulin resistance and metabolic disorders, characterized by decreased fat oxidation in mice, partly due to reduced protein levels of key metabolic regulators or enzymes through acetylation modification, such as CREBH, PPARα, and peroxisome proliferator-activated receptor gamma coactivator 1-α.^98^ Recent studies have shown that the interaction between the transcriptional regulators CREBH and PPARα regulates FAO and lipolysis by controlling the expression of the metabolic hormone FGF21.^99^ However, suppression of the CREBH-PPARα axis leads to hepatic steatosis, induced by the inhibition of mNADK activity following high-fat diet, thus elevating ROS levels.^99^ Furthermore, SIRTs, a class of NAD-dependent nutrient-sensing deacetylases sensitive to ROS, facilitate the degradation of ROS via the proteasome.^98^ Reduced ATP and cyclic adenosine monophosphate levels due to SIRT3 deficiency hinder the CREBH-activated glucagon pathway, exacerbating fasting-induced hepatic steatosis.^100^ This may partly explain why mNADK deficiency increases susceptibility to NASH and hepatocellular carcinoma (Fig. 3).

Further investigation is needed to comprehensively grasp the regulatory mechanisms and molecular functions of mNADK, given that PTMs can disrupt substrate binding and affect enzyme activity, thereby impacting mNADK's functionality.

### MESH1 and NOCT in NADP(H) conversion to NAD(H)

Maintaining precise regulation of NADP(H) and NAD(H) levels is critical for metabolic homeostasis. In addition to cNADK, which is well-characterized to convert NAD^+^ into NADP^+^, two recently identified phosphatases, MESH1^58^ and NOCT,^55^, ^56^, ^57^ play significant roles in converting NADP(H) back into NAD(H). Both enzymes hydrolyze the 2′-phosphate group of NADP(H) to generate NAD(H) and a phosphate.^56^^,^^58^

MESH1 is a cytosolic NADPH phosphatase in mammals, and it shares homology with the bacterial (p)ppGpp hydrolase SpoT.^101^ Under stress conditions, MESH1 dephosphorylates cytoplasmic NADPH to produce NADH, relying on Mn^2+^ as a cofactor.^58^^,^^101^ This process reduces the glutathione which is used to detoxify lipids and prevent cells from ferroptosis.^102^ Previous studies have highlighted that MESH1 promotes ferroptosis, and the expression of MESH1 may increase under conditions that induce ferroptosis, such as erastin treatment or cystine deprivation.^58^

On the other hand, NOCT is considered a circadian gene, with its mRNA distributed widely throughout the body.^103^ It is a member of the CCR4 family of endonuclease-exonuclease-phosphatase enzymes and boasts an evolutionarily conserved leucine zipper-like motif at the N-terminus.^55^ Depending on the translation initiation sites, NOCT can be classified into two isoforms: one is translated from the mitochondrial targeting sequence and transported to the mitochondria; the other is constitutively expressed and remains in the cytoplasm, attaching to the membranes of other organelles via N-terminal glycine myristoylation.^56^^,^^57^ The expression level of mitochondrial NOCT fluctuates rhythmically, peaking during the early dark phase.^56^^,^^57^ This rhythmic cellular localization of NOCT indicates that the circadian clock regulates NADP(H) levels within specific cellular regions at different times.^56^ Changes in mitochondrial NADP(H) levels can have broad metabolic implications. For example, increased mitochondrial NADP(H) may protect NOCT knockout mice from developing hepatic steatosis when on a high-fat diet.^104^ The NADPH concentration near the endoplasmic reticulum is also essential for the activity of cytochrome P450 enzymes anchored to the endoplasmic reticulum.^105^ The N-terminus of NOCT contains a conserved leucine zip-like motif that is vital for maintaining the enzyme's stability and structural integrity, and it regulates the substrate binding flexibility and, consequently, the phosphatase activity.^55^ Deleting the N-terminus or LZ-like helix alters the folding, stability, and conformation of the NOCT protein, ultimately impacting the rate of NADP(H) conversion to NAD(H) and an inorganic phosphate. Additionally, there are other hypotheses regarding NOCT's regulation of NADPH. For instance, NADH produced through the dephosphorylation of NADPH by NOCT might serve as a substrate for the electron transport chain, potentially increasing the spare respiratory capacity and providing more energy for consumption during wakefulness. Moreover, local oxidation may modify the N-terminus of NOCT, affecting its dephosphorylation activity. However, further studies are necessary to understand the precise roles of NOCT in regulating NADPH.

### Regulation of NAD(H) and NADP(H) homeostasis by the circadian clock

NAD^+^ plays a central role in metabolic pathways, influencing cellular redox balance and energy regulation. Its metabolism is closely intertwined with the circadian rhythm, exhibiting a 24-h rhythmicity due to circadian rhythms.^60^^,^^106^, ^107^, ^108^, ^109^, ^110^, ^111^ The conversion of nicotinamide into nicotinamide mononucleotide and subsequently into NAD^+^ is facilitated by nicotinamide phosphoribosyltransferase (NAMPT, the rate-limiting enzyme for the NAD^+^ salvage pathway) and nicotinamide mononucleotide adenylyltransferases, respectively. The circadian clock gene complex, circadian locomotor output cycles kaput (CLOCK), and basic helix-loop-helix ARNT-like protein 1 (BMAL1) regulate NAMPT, activating its promoter every 24 h and enhancing NAD^+^ production.^108^^,^^110^ NAD^+^-dependent histone deacetylase SIRT1 suppresses the circadian transcription of core clock genes such as cryptochrome 1, period 2, and BMAL1 via rhythmic deacetylation; this process may further decrease NAD^+^ levels by inhibiting NAMPT expression.^112^^,^^113^

**Figure 4:**
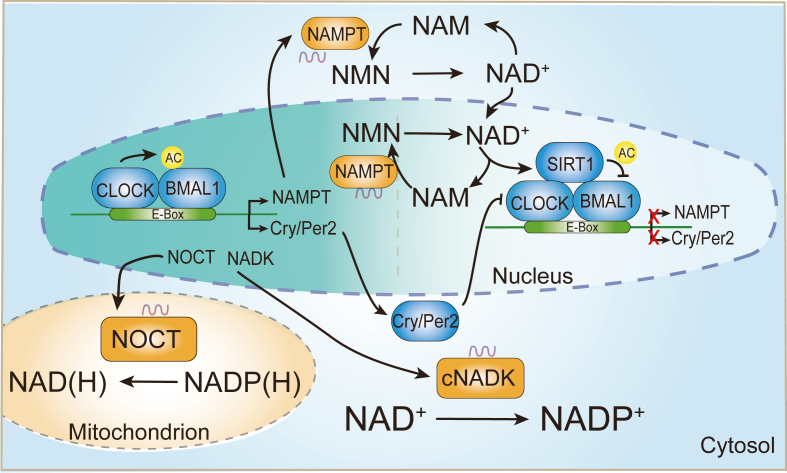
NAD(H) and NADP(H) homeostasis regulated by the circadian clock. Nicotinamide (NAM) is converted to nicotinamide mononucleotide (NMN) by nicotinamide phosphoribosyltransferase (NAMPT), which is further converted to NAD^+^ by nicotinamide mononucleotide adenylyltransferases (NMNAT) in the NAD synthesis salvage pathway. The circadian locomotor output cycles kaput (CLOCK)–basic helix-loop-helix ARNT-like protein 1 (BMAL1) complex periodically activates the NAMPT promoter every 24 h, increasing NAD^+^ production. The NAD^+^-dependent histone deacetylase SIRT1 rhythmically deacetylates and represses the circadian transcription of core clock genes such as cryptochrome 1 (Cry1), period 2 (Per2), and BMAL1, further reducing NAD^+^ levels by inhibiting NAMPT expression. Cytosolic NAD kinase (cNADK) also exhibits an oscillatory rhythmic expression pattern, peaking during early dark stages, suggesting similar oscillatory rhythmicity for NADP^+^ synthesis. Additionally, mitochondrial NOCT, mediating the conversion of NADP(H) into NAD(H), also demonstrates an oscillatory rhythmic expression pattern, peaking during early dark stages.

In mice with a deficient circadian clock (*bmal1*^*−/−*^ or clock^Δ19/Δ19^), intracellular NAD^+^ levels in the liver are significantly reduced.^106^^,^^108^ Conversely, altered NAD^+^ levels can impact the circadian clock, as demonstrated in CD38-deficient mice, where increased NAD^+^ levels lead to abnormal circadian behavior and metabolism (Fig. 4).^114^

Additionally, cNADK exhibits an oscillatory rhythmic expression pattern, peaking during the early dark stage.^110^^,^^111^ Consequently, NADP^+^ synthesis also follows a similar oscillatory rhythmicity. Similarly, mitochondrial NOCT regulates the reversible conversion of NADP(H) into NAD(H) with an oscillatory rhythmic expression pattern, peaking during the early dark stage.^56^^,^^110^ It is crucial to measure the conversion of NADP(H) into NAD(H) in mitochondria to understand the significance of NOCT's oscillatory expression pattern.^56^ However, measuring mitochondrial NAD(H) and NADP(H) levels regulated by circadian rhythms remains challenging (Fig. 4).

### Targeting NAD^+^ and NADP^+^ synthesis in human diseases

The synthesis of NAD^+^ and NADP(H) is critical for a wide range of cellular functions and holds significant implications for human health and disease. NAD^+^ synthesis, regulated by enzymes such as NAMPT and nicotinamide riboside kinase 1/2, plays a key role in various biological processes, including energy metabolism, DNA repair, and gene expression, acting as a cofactor for SIRTs and PARPs.^115^ Dysregulation of NAD^+^ synthesis can disrupt cellular function and has been linked to aging, neurodegenerative diseases, and cancer.^116^, ^117^, ^118^ On the other hand, NADP(H) synthesis, primarily governed by NADKs, is essential for redox balance and the detoxification of ROS, with implications for metabolic disorders, inflammation, oxidative stress-related diseases, and cancer.^119^, ^120^, ^121^ Targeting the synthesis of NAD^+^ and NADP(H) represents a promising and innovative strategy for cancer therapy and the management of various other human diseases.^3^^,^^122^

NAMPT, the key enzyme involved in the salvage pathway of NAD^+^ synthesis, has been found up-regulated in various types of cancer, including COAD,^123^ ovarian cancer,^124^ breast cancer,^125^ and melanoma,^126^ and is associated with poor prognosis.^127^, ^128^, ^129^ Inhibiting NAMPT has been shown to reduce NAD^+^ levels, leading to cancer cell death and impeding tumor growth.^127^ Several NAMPT inhibitors, such as FK866, GMX1778, CHS828,^130^ and OT-82,^124^ have been developed and are currently undergoing clinical trials to assess their efficacy in treating various types of cancer.^131^ Nicotinamide riboside kinase 1/2 are enzymes involved in the alternative NAD^+^ synthesis pathway that phosphorylates nicotinamide riboside to form NAD^+^. Their modulation has been explored as a therapeutic strategy for various diseases, including cancer, neurodegenerative disorders, and metabolic diseases.^132^ Targeting nicotinamide riboside kinase 1/2 has the potential to regulate NAD^+^ levels, thus impacting essential cellular processes such as metabolism, DNA repair, and cellular signaling.^133^

NADKs, through their role in phosphorylating NAD^+^ to NADP^+^, contribute to the synthesis of NADPH, which is essential for various cellular functions, including lipids and nucleotides biosynthesis, maintenance of redox balance, and the detoxification of ROS. Dysregulation of NADKs can potentially lead to metabolic disorders, inflammation, oxidative stress-related diseases, and cancer, making these enzymes attractive targets for therapeutic intervention in various human diseases.^119^^,^^121^

### cNADK in cancer

NADKs, as the key regulators of NADPH levels, have emerged as promising targets for cancer therapy. Aberrant up-regulation of cNADK expression or activity enables tumor cells to overcome restricted redox homeostasis and acquire the necessary nucleotide precursors by elevating NADPH levels, which are crucial for tumorigenesis and cancer development.^2^^,^^93^^,^^94^ Moreover, NADPH acts as a cofactor for generating D-2-hydroxyglutarate, an oncogenic metabolite derived from α-ketoglutarate prevalent in isocitrate dehydrogenase mutant cancers.^134^^,^^135^ The Cancer Genome Atlas data have revealed that cNADK is overexpressed in various tumors and correlates negatively with patient prognosis.^136^^,^^137^ Additionally, the gain-of-function I90F mutant in cNADK is positively correlated with the progression of PDAC.^95^ Silencing cNADK reduces NADPH pools and inhibits cancer cell growth in diffuse large B-cell lymphoma and COAD.^138^ In metastatic breast cancer cells, increased cNADK levels enhance metastatic ability through epigenetic regulation of the cNADK promoter by the histone H3.3 variant.^137^ In Notch1-driven Jurkat cells, cNADK silencing leads to significantly elevated ROS levels, making cNADK a potential therapeutic target for Notch1-driven T-ALL.^139^ These findings highlight the therapeutic potential of cNADK, both in its wild-type and specific mutant variants, for cancer treatment.

Growth factor-stimulated PI3K-Akt signaling promotes cellular anabolism either by the direct phosphorylation of key metabolic enzymes or through transcriptional regulation.^88^ These key anabolic processes, including nucleotide, lipid, and amino acid synthesis, require abundant reducing equivalents in the form of NADPH. To meet this demand, AKT directly phosphorylates cNADK, enhancing its enzyme activity and increasing NADP(H) production. Activated PI3K-AKT signaling is a prominent feature of many tumors.^87^ AKT phosphorylates cNADK at serine 44, 46, and 48, releasing the N-terminal autoinhibitory conformation to boost the activity of cNADK, thus facilitating NADP^+^ production and supporting rapid tumor proliferation.^65^^,^^87^

The gain-of-function mutation (G12V) in KRAS, a key oncogenic driver in various tumors like PDAC and COAD, alters tumor metabolic flux.^91^^,^^92^^,^^140^ Through PKC-mediated phosphorylation, activated KRAS can enhance cNADK activity by phosphorylating serine 46 and 64, thus promoting NADP^+^ production and contributing to PDAC tumorigenesis and progression.^89^^,^^90^

**Figure 5:**
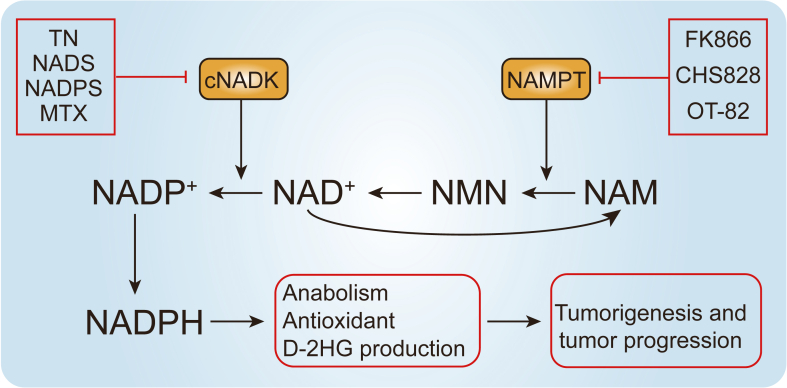
The role of cytosolic NAD kinase (cNADK) and nicotinamide phosphoribosyltransferase (NAMPT) in cancer. NAMPT, a crucial enzyme in the NAD^+^ synthesis salvage pathway, converts nicotinamide (NAM) to nicotinamide mononucleotide (NMN), which is then adenylated to NAD^+^. cNADK is responsible for phosphorylating NAD^+^ to NADP^+^. NADP^+^ is then reduced to NADPH, crucial for maintaining cellular redox homeostasis and participating in multiple biosynthetic pathways. Abnormal up-regulation of cNADK or NAMPT, either in expression or enzymatic activity, enables tumor cells to bypass redox constraints and nucleotide precursor availability. This leads to increased NADPH production, promoting tumorigenesis and cancer progression. Inhibitors targeting NADKs (such as thionicotinamide (TN), NADS, NADPS, and methotrexate (MTX)) and NAMPT (such as FK866, CHS828, and OT-82) could potentially mitigate tumorigenesis and tumor development by decreasing NADPH synthesis.

Given the critical role of cNADK in cancer, targeting this enzyme has gained attention in cancer therapy.^136^ Thionicotinamide, a dual inhibitor of cNADK and G6PD, reduces intracellular NADPH, leading to an increased ROS.^138^^,^^141^^,^^142^ This depletion of NADPH escalates oxidative stress in cancer cells, reduces the supply of nucleic acid essential for DNA replication and also inhibits other NADPH-dependent processes, including fatty acid synthesis.^94^^,^^138^ In xenograft mouse models of COAD and diffuse large B-cell lymphoma, thionicotinamide treatment inhibited tumor development and sensitized cells to various chemotherapeutic drugs, including methotrexate, gemcitabine, docetaxel, and irinotecan.^138^ Moreover, methotrexate, a first-line anti-rheumatoid arthritis drug and anticancer chemotherapy agent, was recently confirmed as a cNADK inhibitor.^53^^,^^72^ Methotrexate treatment rapidly decreased cNADK activity in the normal liver.^53^ Nicotinamide riboside can be used to mitigate the hepatotoxicity caused by methotrexate (Fig. 5).

## NADKs in metabolic diseases

### cNADK in metabolic diseases

Hepatic NAD^+^ deficiency is a target for therapeutic intervention in NASH.^143^ Interestingly, studies have suggested that cNADK levels are increased in liver cells of NASH patients, concomitant with reduced NAD^+^ levels.^86^ Notably, cNADK has been identified as a target for miR-690, and up-regulation of miR-690 leads to reduced cNADK levels.^84^^,^^86^ This miR-690-mediated inhibition of cNADK has shown promising results in mitigating fibrosis and inflammation, thus slowing down disease progression in NASH models.^84^^,^^86^ Kupffer cells, the liver's resident macrophages, express high levels of endogenous miR-690, which enhances their autoproliferation by inhibiting cNADK.^85^^,^^86^ Moreover, miR-690 can be shuttled to other nearby hepatic cells or hepatic stellate cells through secreted exosomes, providing an additional mechanism for reducing fibrosis and inflammation in NASH models (Fig. 2).^86^

### mNADK in metabolic diseases

**Figure 6:**
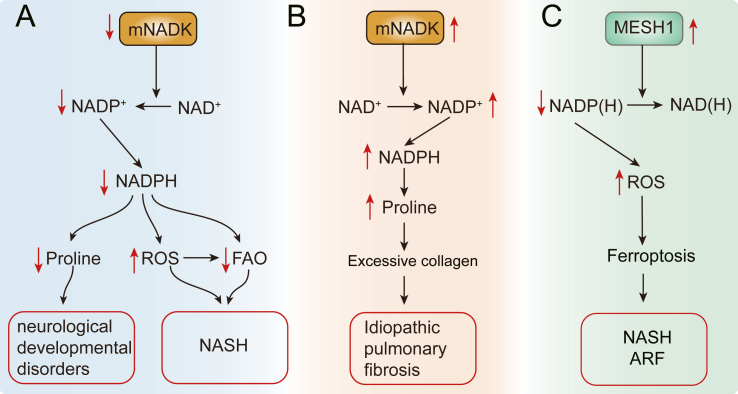
The roles of mNADK and MESH1 in metabolic disorders. **(A)** The role of mNADK in neurological developmental disorders and nonalcoholic steatohepatitis (NASH). Down-regulated mNADK results in lower NADPH levels, influencing proline synthesis and potentially contributing to neurological developmental disorders. Diminished NADPH also results in increased ROS levels and impaired fatty acid oxidation (FAO), contributing to NASH progression. **(B)** The role of mNADK in idiopathic pulmonary fibrosis. Elevated mNADK expression induces excessive collagen deposition, exacerbating idiopathic pulmonary fibrosis. **(C)** The role of MESH1 in NASH and acute renal failure (ARF). Overexpression of MESH1 decreases cellular NADPH levels and increases ROS, sensitizing cells to ferroptosis, and promoting the occurrence of NASH and ARF.

Conversely, mNADK plays a critical role in preventing severe hepatic steatosis and hypertriglyceridemia.^71^^,^^72^ Knocking out mNADK in hepatocytes results in lower NADPH levels and increased ROS levels, leading to impaired downstream gene transcription, such as CREBH and PPAR, causing defects in FAO.^71^ The combination of elevated ROS and defective FAO contributes to the progression of NASH (Fig. 6A).

Additionally, the loss of mNADK results in increased levels of lysine and carnitine in the plasma of mice or some patients, disrupting proline biosynthesis and subsequent cell proliferation and contributing to various diseases.^23^^,^^24^^,^^72^^,^^83^ Proline deficiency, possibly caused by mNADK mutations discovered in a subset of patients, has been implicated in neurological developmental disorders (Fig. 6A).^83^ Proline levels also affect collagen production in patients with osteosarcoma and chondrosarcoma, and proline supplementation has been shown to compensate for decreased collagen production in fibroblasts deficient in mNADK.^23^ However, excessive proline can also be detrimental. For instance, idiopathic pulmonary fibrosis is associated with elevated mNADK expression and excessive collagen deposition (Fig. 6B).^23^ Therefore, maintaining a balance of mNADK and proline metabolism is crucial to health.

### MESH1 and NOCT in human diseases

Dysregulation of MESH1, a metazoan homolog of the bacterial SpoT protein, plays a significant role in the progression of human diseases, particularly in conditions related to cancer and oxidative stress. MESH1 knockdown triggers a set of survival mechanisms resembling the bacterial stringent response, including cell proliferation arrest, alterations in the transcriptome, and induction of endoplasmic reticulum stress pathways.^101^^,^^144^ One critical finding is MESH1's impact on TAZ repression, a crucial effector of the Hippo pathway that regulates cell proliferation and organ size.^144^ MESH1 also modulates ferroptosis by controlling the degradation of NADPH, a central metabolite in redox homeostasis.^58^ Consequently, MESH1 dysregulation can potentially contribute to various pathological conditions associated with dysregulated ferroptosis, such as neurotoxicity and acute renal, hepatic, and cardiac injuries (Fig. 6C).^58^ Despite some gaps in our current knowledge, MESH1 represents a potential therapeutic target for stress-related diseases and cancer.

Dysregulation of NOCT, a clock-controlled protein, and NADPH phosphatase, could potentially influence several aspects of human health and disease. Disruptions in NOCT function may lead to disturbances in metabolic and circadian rhythms, potentially contributing to metabolic disorders such as obesity, diabetes, and nonalcoholic fatty liver disease.^57^^,^^145^ This is likely due to the role of NOCT in energy metabolism, body weight regulation, and insulin sensing.^57^ Moreover, NOCT dysregulation may affect cellular redox status, as it regulates protein glutathionylation, a process that protects proteins from oxidative damage.^56^ Given NOCT's role in managing NADPH levels, which in turn influence ROS levels, its dysregulation could impact diseases related to ROS signaling and oxidative damage.^56^ These results suggest that NOCT dysregulation could enhance susceptibility to diseases related to oxidative stress. Additionally, NOCT dysregulation could contribute to bone-related diseases such as osteoporosis and neurological disorders due to its involvement in osteoblast differentiation and regulation of neuronal genes, respectively.^145^ More research is necessary to fully understand the precise roles of NOCT and its implications for human diseases.

## Conclusion and prospects

Maintaining the homeostasis of NAD(H) and NADP(H) is crucial for cell proliferation and metabolism, with NAD^+^ governing redox reactions (catabolism) and NADPH driving cell anabolism. NADKs are currently the only known enzymes facilitating the conversion of NAD^+^ to NADP^+^, while MESH1 and NOCT have recently been identified as the two phosphatases capable of reversing NADP(H) to NAD(H). The interconversion between NAD(H) and NADP(H), regulated by NADKs, NOCT, and MESH1, plays a crucial role in cellular physiological and pathological activities. Abnormal regulation of these enzymes is implicated in various diseases. Therefore, investigating the relationship between the abundance/activity of NADKs/NOCT/MESH1, and the cellular levels of NAD(H)/NADP(H) under varying conditions may shed light on their regulatory mechanisms and networks.

Despite significant recent advances in our understanding of NAD(H) and NADP(H) homeostatic regulation, many questions remain unanswered and require systematic exploration in the future. For instance, how do organisms sense the balance of NAD(H) and NADP(H) pools? Besides the recently discovered SLC25A51,^146^, ^147^, ^148^ which molecules mediate NAD^+^ transport from the cytoplasm to the mitochondria, considering cell membranes are impermeable? Are there other transporter proteins facilitating the transport of NAD(H) and NADP(H)? Do eukaryotes possess other organelle-specific NADKs, such as those for peroxisomes and the endoplasmic reticulum? How does the regulation of NADKs vary across different tissues and under diverse physiological or pathological conditions? Are there additional regulatory mechanisms involving PTMs? What are the molecular mechanisms governing the oscillatory rhythms of cNADK and mitochondrial NOCT in the circadian rhythm?

As key enzymes regulating NAD(H) and NADP(H) homeostasis, NADKs have been proposed as drug targets for disease treatment. However, with the recent discovery of counter-responsive enzymes MESH1 and NOCT, modulating NADPH supply in reverse is now feasible. Additionally, considering the rhythmic regulation of NOCT, elucidating its regulatory mechanisms, and developing targeted therapy might represent a novel approach to cancer treatment.

## Author contributions

Conceptualization: Huadong Pei; Writing-review and editing: Luojun Chen, Xiaoke Xing, Pingfeng Zhang, and Lulu Chen; Supervision: Huadong Pei. All authors read and approved the final manuscript.

## Conflict of interests

The authors declare that they have no competing interests.

## References

[1] Covarrubias A.J., Perrone R., Grozio A., Verdin E. (2021). NAD^+^ metabolism and its roles in cellular processes during ageing. Nat Rev Mol Cell Biol.

[2] Ju H.Q., Lin J.F., Tian T., Xie D., Xu R.H. (2020). NADPH homeostasis in cancer: functions, mechanisms and therapeutic implications. Signal Transduct Targeted Ther.

[3] Zapata-Pérez R., Wanders R.J.A., van Karnebeek C.D.M., Houtkooper R.H. (2021). NAD^+^ homeostasis in human health and disease. EMBO Mol Med.

[4] Xiao W., Wang R.S., Handy D.E., Loscalzo J. (2018). NAD(H) and NADP(H) redox couples and cellular energy metabolism. Antioxidants Redox Signal.

[5] Navas L.E., Carnero A. (2021). NAD^+^ metabolism, stemness, the immune response, and cancer. Signal Transduct Targeted Ther.

[6] Houtkooper R.H., Pirinen E., Auwerx J. (2012). Sirtuins as regulators of metabolism and healthspan. Nat Rev Mol Cell Biol.

[7] Gupte R., Liu Z., Kraus W.L. (2017). PARPs and ADP-ribosylation: recent advances linking molecular functions to biological outcomes. Genes Dev.

[8] Ke Y., Zhang J., Lv X., Zeng X., Ba X. (2019). Novel insights into PARPs in gene expression: regulation of RNA metabolism. Cell Mol Life Sci.

[9] Munnur D., Bartlett E., Mikolčević P. (2019). Reversible ADP-ribosylation of RNA. Nucleic Acids Res.

[10] Ray S., Abugable A.A., Parker J. (2022). A mechanism for oxidative damage repair at gene regulatory elements. Nature.

[11] Bilokapic S., Suskiewicz M.J., Ahel I., Halic M. (2020). Bridging of DNA breaks activates PARP2-HPF_1_ to modify chromatin. Nature.

[12] Suskiewicz M.J., Zobel F., Ogden T.E.H. (2020). HPF_1_ completes the PARP active site for DNA damage-induced ADP-ribosylation. Nature.

[13] Takasawa S. (2022). CD38-cyclic ADP-ribose signal system in physiology, biochemistry, and pathophysiology. Int J Mol Sci.

[14] Lee H.C. (1997). Mechanisms of calcium signaling by cyclic ADP-ribose and NAADP. Physiol Rev.

[15] Malavasi F., Deaglio S., Funaro A. (2008). Evolution and function of the ADP ribosyl cyclase/*CD38* gene family in physiology and pathology. Physiol Rev.

[16] Partida-Sánchez S., Cockayne D.A., Monard S. (2001). Cyclic ADP-ribose production by CD38 regulates intracellular calcium release, extracellular calcium influx, and chemotaxis in neutrophils and is required for bacterial clearance *in vivo*. Nat Med.

[17] Partida-Sánchez S., Goodrich S., Kusser K., Oppenheimer N., Randall T.D., Lund F.E. (2004). Regulation of dendritic cell trafficking by the ADP-ribosyl cyclase CD38: impact on the development of humoral immunity. Immunity.

[18] Lupu R., Menendez J.A. (2006). Pharmacological inhibitors of fatty acid synthase (FASN): catalyzed endogenous fatty acid biogenesis: a new family of anti-cancer agents?. Curr Pharmaceut Biotechnol.

[19] Luo J., Yang H., Song B.L. (2020). Mechanisms and regulation of cholesterol homeostasis. Nat Rev Mol Cell Biol.

[20] Moreno-Sánchez R., Gallardo-Pérez J.C., Rodríguez-Enríquez S., Saavedra E., Marín-Hernández Á. (2017). Control of the NADPH supply for oxidative stress handling in cancer cells. Free Radic Biol Med.

[21] Palde P.B., Carroll K.S. (2015). A universal entropy-driven mechanism for thioredoxin-target recognition. Proc Natl Acad Sci U S A.

[22] Pandey A.V., Flück C.E. (2013). NADPH P450 oxidoreductase: structure, function, and pathology of diseases. Pharmacol Ther.

[23] Zhu J., Schwörer S., Berisa M. (2021). Mitochondrial NADP(H) generation is essential for proline biosynthesis. Science.

[24] Tran D.H., Kesavan R., Rion H. (2021). Mitochondrial NADP^+^ is essential for proline biosynthesis during cell growth. Nat Metab.

[25] Zheng Y., Lin T.Y., Lee G. (2018). Mitochondrial one-carbon pathway supports cytosolic folate integrity in cancer cells. Cell.

[26] Cory J.G., Sato A. (1983). Regulation of ribonucleotide reductase activity in mammalian cells. Mol Cell Biochem.

[27] Panday A., Sahoo M.K., Osorio D., Batra S. (2015). NADPH oxidases: an overview from structure to innate immunity-associated pathologies. Cell Mol Immunol.

[28] Szanto I. (2022). NADPH oxidase 4 (NOX4) in cancer: linking redox signals to oncogenic metabolic adaptation. Int J Mol Sci.

[29] Winterbourn C.C., Kettle A.J., Hampton M.B. (2016). Reactive oxygen species and neutrophil function. Annu Rev Biochem.

[30] Badawy A.A.B. (2017). Kynurenine pathway of tryptophan metabolism: regulatory and functional aspects. Int J Tryptophan Res.

[31] Braidy N., Berg J., Clement J. (2019). Role of nicotinamide adenine dinucleotide and related precursors as therapeutic targets for age-related degenerative diseases: rationale, biochemistry, pharmacokinetics, and outcomes. Antioxidants Redox Signal.

[32] Evans J., Wang T.C., Heyes M.P., Markey S.P. (2002). LC/MS analysis of NAD biosynthesis using stable isotope pyridine precursors. Anal Biochem.

[33] Xie N., Zhang L., Gao W. (2020). NAD^+^ metabolism: pathophysiologic mechanisms and therapeutic potential. Signal Transduct Targeted Ther.

[34] Reitman Z.J., Yan H. (2010). Isocitrate dehydrogenase 1 and 2 mutations in cancer: alterations at a crossroads of cellular metabolism. J Natl Cancer Inst.

[35] Asadi Shahmirzadi A., Edgar D., Liao C.Y. (2020). Alpha-ketoglutarate, an endogenous metabolite, extends lifespan and compresses morbidity in aging mice. Cell Metabol.

[36] Minárik P., Tomásková N., Kollárová M., Antalík M. (2002). Malate dehydrogenases: structure and function. Gen Physiol Biophys.

[37] Yang S.Y., He X.Y., Schulz H. (2005). 3-Hydroxyacyl-CoA dehydrogenase and short chain 3-hydroxyacyl-CoA dehydrogenase in human health and disease. FEBS J.

[38] Gu H., Chen C., Hao X. (2020). MDH1-mediated malate-aspartate NADH shuttle maintains the activity levels of fetal liver hematopoietic stem cells. Blood.

[39] Wang Y., Stancliffe E., Fowle-Grider R. (2022). Saturation of the mitochondrial NADH shuttles drives aerobic glycolysis in proliferating cells. Mol Cell.

[40] Granchi C., Bertini S., Macchia M., Minutolo F. (2010). Inhibitors of lactate dehydrogenase isoforms and their therapeutic potentials. Curr Med Chem.

[41] Fan J., Ye J., Kamphorst J.J., Shlomi T., Thompson C.B., Rabinowitz J.D. (2014). Quantitative flux analysis reveals folate-dependent NADPH production. Nature.

[42] Jiang P., Du W., Mancuso A., Wellen K.E., Yang X. (2013). Reciprocal regulation of p53 and malic enzymes modulates metabolism and senescence. Nature.

[43] Lu Y.X., Ju H.Q., Liu Z.X. (2019). Correction: ME1 regulates NADPH homeostasis to promote gastric cancer growth and metastasis. Cancer Res.

[44] Al-Khallaf H. (2017). Isocitrate dehydrogenases in physiology and cancer: biochemical and molecular insight. Cell Biosci.

[45] Cai W.F., Zhang C., Wu Y.Q. (2018). Glutaminase GLS1 senses glutamine availability in a non-enzymatic manner triggering mitochondrial fusion. Cell Res.

[46] Jiang P., Du W., Wu M. (2014). Regulation of the pentose phosphate pathway in cancer. Protein Cell.

[47] Chen L., Zhang Z., Hoshino A. (2019). NADPH production by the oxidative pentose-phosphate pathway supports folate metabolism. Nat Metab.

[48] Wamelink M.M., Struys E.A., Jakobs C. (2008). The biochemistry, metabolism and inherited defects of the pentose phosphate pathway: a review. J Inherit Metab Dis.

[49] Ralto K.M., Rhee E.P., Parikh S.M. (2020). NAD^+^ homeostasis in renal health and disease. Nat Rev Nephrol.

[50] Krupenko N.I., Holmes R.S., Tsybovsky Y., Krupenko S.A. (2015). Aldehyde dehydrogenase homologous folate enzymes: evolutionary switch between cytoplasmic and mitochondrial localization. Chem Biol Interact.

[51] Nilsson R., Jain M., Madhusudhan N. (2014). Metabolic enzyme expression highlights a key role for MTHFD2 and the mitochondrial folate pathway in cancer. Nat Commun.

[52] Ward N.P., Kang Y.P., Falzone A., Boyle T.A., DeNicola G.M. (2020). Nicotinamide nucleotide transhydrogenase regulates mitochondrial metabolism in NSCLC through maintenance of Fe-S protein function. J Exp Med.

[53] Mhatre R.M., Saslaw L.D., Waravdekar V.S. (1967). Effect of methotrexate on NAD kinase activity in leukaemic mice. Nature.

[54] Liu L., Su X., Quinn W.J. (2018). Quantitative analysis of NAD synthesis-breakdown fluxes. Cell Metabol.

[55] Wickramaratne A.C., Li L., Hopkins J.B., Joachimiak L.A., Green C.B. (2022). The disordered amino terminus of the circadian enzyme nocturnin modulates its NADP(H) phosphatase activity by changing protein dynamics. Biochemistry.

[56] Laothamatas I., Gao P., Wickramaratne A. (2020). Spatiotemporal regulation of NADP(H) phosphatase Nocturnin and its role in oxidative stress response. Proc Natl Acad Sci USA.

[57] Estrella M.A., Du J., Chen L. (2019). The metabolites NADP^+^ and NADPH are the targets of the circadian protein Nocturnin (Curled). Nat Commun.

[58] Ding C.C., Rose J., Sun T. (2020). MESH1 is a cytosolic NADPH phosphatase that regulates ferroptosis. Nat Metab.

[59] Wang T.A., Yu Y.V., Govindaiah G. (2012). Circadian rhythm of redox state regulates excitability in suprachiasmatic nucleus neurons. Science.

[60] Reinke H., Asher G. (2019). Crosstalk between metabolism and circadian clocks. Nat Rev Mol Cell Biol.

[61] Levine D.C., Ramsey K.M., Bass J. (2022). Circadian NAD(P)(H) cycles in cell metabolism. Semin Cell Dev Biol.

[62] Yoshino J. (2013). Importance of NAMPT-mediated NAD-biosynthesis and NAD-dependent deacetylase SIRT1 in the crosstalk between circadian rhythm and metabolism. Nihon Rinsho.

[63] Levine D.C., Kuo H.Y., Hong H.K. (2021). NADH inhibition of SIRT1 links energy state to transcription during time-restricted feeding. Nat Metab.

[64] Putker M., Crosby P., Feeney K.A. (2018). Mammalian circadian period, but not phase and amplitude, is robust against redox and metabolic perturbations. Antioxidants Redox Signal.

[65] Hoxhaj G., Ben-Sahra I., Lockwood S.E. (2019). Direct stimulation of NADP^+^ synthesis through Akt-mediated phosphorylation of NAD kinase. Science.

[66] Shi F., Li Y., Li Y., Wang X. (2009). Molecular properties, functions, and potential applications of NAD kinases. Acta Biochim Biophys Sin.

[67] Ohashi K., Kawai S., Murata K. (2012). Identification and characterization of a human mitochondrial NAD kinase. Nat Commun.

[68] Lerner F., Niere M., Ludwig A., Ziegler M. (2001). Structural and functional characterization of human NAD kinase. Biochem Biophys Res Commun.

[69] Mary C., Soflaee M.H., Kesavan R. (2022). Crystal structure of human NADK2 reveals a dimeric organization and active site occlusion by lysine acetylation. Mol Cell.

[70] Du J., Estrella M., Solorio-Kirpichyan K., Jeffrey P.D., Korennykh A. (2022). Structure of human NADK2 reveals atypical assembly and regulation of NAD kinases from animal mitochondria. Proc Natl Acad Sci U S A.

[71] Kim H., Fu Z., Yang Z. (2022). The mitochondrial NAD kinase functions as a major metabolic regulator upon increased energy demand. Mol Metabol.

[72] Zhang K., Kim H., Fu Z. (2018). Deficiency of the mitochondrial NAD kinase causes stress-induced hepatic steatosis in mice. Gastroenterology.

[73] Bieganowski P., Seidle H.F., Wojcik M., Brenner C. (2006). Synthetic lethal and biochemical analyses of NAD and NADH kinases in *Saccharomyces cerevisiae* establish separation of cellular functions. J Biol Chem.

[74] Oka S.I., Titus A.S., Zablocki D., Sadoshima J. (2023). Molecular properties and regulation of NAD^+^ kinase (NADK). Redox Biol.

[75] Ivarsson R., Quintens R., Dejonghe S. (2005). Redox control of exocytosis: regulatory role of NADPH, thioredoxin, and glutaredoxin. Diabetes.

[76] Gray J.P., Alavian K.N., Jonas E.A., Heart E.A. (2012). NAD kinase regulates the size of the NADPH pool and insulin secretion in pancreatic β-cells. Am J Physiol Endocrinol Metab.

[77] Xu M., Ding L., Liang J. (2021). NAD kinase sustains lipogenesis and mitochondrial metabolismthrough fatty acid synthesis. Cell Rep.

[78] Ritterhoff J., Young S., Villet O. (2020). Metabolic remodeling promotes cardiac hypertrophy by directing glucose to aspartate biosynthesis. Circ Res.

[79] Matsuzawa-Nagata N., Takamura T., Ando H. (2008). Increased oxidative stress precedes the onset of high-fat diet-induced insulin resistance and obesity. Metabolism.

[80] De Ingeniis J., Ratnikov B., Richardson A.D. (2012). Functional specialization in proline biosynthesis of melanoma. PLoS One.

[81] Dickinson M.E., Flenniken A.M., Ji X. (2016). High-throughput discovery of novel developmental phenotypes. Nature.

[82] Pomerantz D.J., Ferdinandusse S., Cogan J. (2018). Clinical heterogeneity of mitochondrial NAD kinase deficiency caused by a NADK2 start loss variant. Am J Med Genet.

[83] Houten S.M., Denis S., te Brinke H. (2014). Mitochondrial NADP(H) deficiency due to a mutation in NADK2 causes dienoyl-CoA reductase deficiency with hyperlysinemia. Hum Mol Genet.

[84] Ying W., Gao H., Dos Reis F.C.G. (2021). miR-690, an exosomal-derived miRNA from M2-polarized macrophages, improves insulin sensitivity in obese mice. Cell Metabol.

[85] Love N.R., Pollak N., Dölle C. (2015). NAD kinase controls animal NADP biosynthesis and is modulated via evolutionarily divergent calmodulin-dependent mechanisms. Proc Natl Acad Sci U S A.

[86] Gao H., Jin Z., Bandyopadhyay G. (2022). miR-690 treatment causes decreased fibrosis and steatosis and restores specific Kupffer cell functions in NASH. Cell Metabol.

[87] Hoxhaj G., Manning B.D. (2020). The PI3K-AKT network at the interface of oncogenic signalling and cancer metabolism. Nat Rev Cancer.

[88] Hopkins B.D., Goncalves M.D., Cantley L.C. (2020). Insulin-PI3K signalling: an evolutionarily insulated metabolic driver of cancer. Nat Rev Endocrinol.

[89] Rabani R., Cossette C., Graham F., Powell W.S. (2020). Protein kinase C activates NAD kinase in human neutrophils. Free Radic Biol Med.

[90] Schild T., McReynolds M.R., Shea C. (2021). NADK is activated by oncogenic signaling to sustain pancreatic ductal adenocarcinoma. Cell Rep.

[91] Cao L., Huang C., Cui Zhou D. (2021). Proteogenomic characterization of pancreatic ductal adenocarcinoma. Cell.

[92] Cancer Genome Atlas Network (2012). Comprehensive molecular characterization of human colon and rectal cancer. Nature.

[93] Zhang Y., Xu Y., Lu W. (2022). G6PD-mediated increase in *de novo* NADP^+^ biosynthesis promotes antioxidant defense and tumor metastasis. Sci Adv.

[94] Zhang Y., Xu Y., Lu W. (2021). Upregulation of antioxidant capacity and nucleotide precursor availability suffices for oncogenic transformation. Cell Metabol.

[95] Tsang Y.H., Dogruluk T., Tedeschi P.M. (2016). Functional annotation of rare gene aberration drivers of pancreatic cancer. Nat Commun.

[96] Zhang R. (2013). MNADK, a novel liver-enriched mitochondrion-localized NAD kinase. Biol Open.

[97] Kawabata Y., Murata K., Kawai S. (2015). Significance of *Ser*-188 in human mitochondrial NAD kinase as determined by phosphomimetic and phosphoresistant amino-acid substitutions. Biochem Biophys Res Commun.

[98] Caito S., Rajendrasozhan S., Cook S. (2010). SIRT1 is a redox-sensitive deacetylase that is post-translationally modified by oxidants and carbonyl stress. Faseb J.

[99] Kim H., Mendez R., Zheng Z. (2014). Liver-enriched transcription factor CREBH interacts with peroxisome proliferator-activated receptor α to regulate metabolic hormone FGF_21_. Endocrinology.

[100] Ahn B.H., Kim H.S., Song S. (2008). A role for the mitochondrial deacetylase Sirt3 in regulating energy homeostasis. Proc Natl Acad Sci U S A.

[101] Sun D., Lee G., Lee J.H. (2010). A metazoan ortholog of SpoT hydrolyzes ppGpp and functions in starvation responses. Nat Struct Mol Biol.

[102] Liu C.C., Gebicki J.M. (2012). Intracellular GSH and ascorbate inhibit radical-induced protein chain peroxidation in HL-60 cells. Free Radic Biol Med.

[103] Stubblefield J.J., Gao P., Kilaru G., Mukadam B., Terrien J., Green C.B. (2018). Temporal control of metabolic amplitude by nocturnin. Cell Rep.

[104] Green C.B., Douris N., Kojima S. (2007). Loss of Nocturnin, a circadian deadenylase, confers resistance to hepatic steatosis and diet-induced obesity. Proc Natl Acad Sci U S A.

[105] Neve E.P.A., Ingelman-Sundberg M. (2008). Intracellular transport and localization of microsomal cytochrome P450. Anal Bioanal Chem.

[106] Nakahata Y., Sahar S., Astarita G., Kaluzova M., Sassone-Corsi P. (2009). Circadian control of the NAD^+^ salvage pathway by CLOCK-SIRT1. Science.

[107] Rutter J., Reick M., Wu L.C., McKnight S.L. (2001). Regulation of clock and NPAS2 DNA binding by the redox state of NAD cofactors. Science.

[108] Ramsey K.M., Yoshino J., Brace C.S. (2009). Circadian clock feedback cycle through NAMPT-mediated NAD^+^ biosynthesis. Science.

[109] Bellet M.M., Nakahata Y., Boudjelal M. (2013). Pharmacological modulation of circadian rhythms by synthetic activators of the deacetylase SIRT1. Proc Natl Acad Sci U S A.

[110] Koronowski K.B., Kinouchi K., Welz P.S. (2019). Defining the independence of the liver circadian clock. Cell.

[111] Laval-Martin D.L., Carré I.A., Barbera S.J., Edmunds L.N. (1990). Circadian variations in the affinities of NAD kinase and NADP phosphatase for their substrates, NAD^+^ and NADP^+^, in dividing and nondividing cells of the achlorophyllous ZC mutant of *Euglena gracilis* Klebs (strain Z). Chronobiol Int.

[112] Asher G., Gatfield D., Stratmann M. (2008). SIRT1 regulates circadian clock gene expression through PER2 deacetylation. Cell.

[113] Nakahata Y., Kaluzova M., Grimaldi B. (2008). The NAD^+^-dependent deacetylase SIRT1 modulates CLOCK-mediated chromatin remodeling and circadian control. Cell.

[114] Sahar S., Nin V., Barbosa M.T., Chini E.N., Sassone-Corsi P. (2011). Altered behavioral and metabolic circadian rhythms in mice with disrupted NAD^+^ oscillation. Aging.

[115] Audrito V., Messana V.G., Deaglio S. (2020). NAMPT and NAPRT: two metabolic enzymes with key roles in inflammation. Front Oncol.

[116] Garten A., Petzold S., Körner A., Imai S.I., Kiess W. (2009). Nampt: linking NAD biology, metabolism and cancer. Trends Endocrinol Metabol.

[117] Fletcher R.S., Ratajczak J., Doig C.L. (2017). Nicotinamide riboside kinases display redundancy in mediating nicotinamide mononucleotide and nicotinamide riboside metabolism in skeletal muscle cells. Mol Metabol.

[118] Bieganowski P., Brenner C. (2004). Discoveries of nicotinamide riboside as a nutrient and conserved *NRK* genes establish a Preiss-Handler independent route to NAD^+^ in fungi and humans. Cell.

[119] Pollak N., Dölle C., Ziegler M. (2007). The power to reduce: pyridine nucleotides: small molecules with a multitude of functions. Biochem J.

[120] Ying W. (2008). NAD^+^/NADH and NADP^+^/NADPH in cellular functions and cell death: regulation and biological consequences. Antioxidants Redox Signal.

[121] Zhang T., Berrocal J.G., Frizzell K.M. (2009). Enzymes in the NAD^+^ salvage pathway regulate SIRT1 activity at target gene promoters. J Biol Chem.

[122] Yaku K., Okabe K., Hikosaka K., Nakagawa T. (2018). NAD metabolism in cancer therapeutics. Front Oncol.

[123] Xie H., Lei Y., Mao Y. (2022). FK866 inhibits colorectal cancer metastasis by reducing NAD^+^ levels in cancer-associated fibroblasts. Genes Genomics.

[124] Wei Y., Xiang H., Zhang W. (2022). Review of various NAMPT inhibitors for the treatment of cancer. Front Pharmacol.

[125] Sharif T., Ahn D.G., Liu R.Z. (2016). The NAD^+^ salvage pathway modulates cancer cell viability via p73. Cell Death Differ.

[126] Galli U., Travelli C., Massarotti A. (2013). Medicinal chemistry of nicotinamide phosphoribosyltransferase (NAMPT) inhibitors. J Med Chem.

[127] Garten A., Schuster S., Penke M., Gorski T., de Giorgis T., Kiess W. (2015). Physiological and pathophysiological roles of NAMPT and NAD metabolism. Nat Rev Endocrinol.

[128] Bi T.Q., Che X.M., Liao X.H. (2011). Overexpression of Nampt in gastric cancer and chemopotentiating effects of the Nampt inhibitor FK866 in combination with fluorouracil. Oncol Rep.

[129] Heske C.M. (2019). Beyond energy metabolism: exploiting the additional roles of NAMPT for cancer therapy. Front Oncol.

[130] Fratta S., Biniecka P., Moreno-Vargas A.J. (2023). Synthesis and structure-activity relationship of new nicotinamide phosphoribosyltransferase inhibitors with antitumor activity on solid and haematological cancer. Eur J Med Chem.

[131] Holen K., Saltz L.B., Hollywood E., Burk K., Hanauske A.R. (2008). The pharmacokinetics, toxicities, and biologic effects of FK866, a nicotinamide adenine dinucleotide biosynthesis inhibitor. Invest N Drugs.

[132] Bogan K.L., Brenner C. (2008). Nicotinic acid, nicotinamide, and nicotinamide riboside: a molecular evaluation of NAD+ precursor vitamins in human nutrition. Annu Rev Nutr.

[133] Cantó C., Menzies K.J., Auwerx J. (2015). NAD^+^ metabolism and the control of energy homeostasis: a balancing act between mitochondria and the nucleus. Cell Metabol.

[134] Zhao M., Yao P., Mao Y. (2022). Malic enzyme 2 maintains protein stability of mutant p53 through 2-hydroxyglutarate. Nat Metab.

[135] Notarangelo G., Spinelli J.B., Perez E.M. (2022). Oncometabolite d-2HG alters T cell metabolism to impair CD8^+^ T cell function. Science.

[136] Tedeschi P.M., Bansal N., Kerrigan J.E., Abali E.E., Scotto K.W., Bertino J.R. (2016). NAD^+^ kinase as a therapeutic target in cancer. Clin Cancer Res.

[137] Ilter D., Schild T., Ward N.P. (2022). NADK upregulation is an essential metabolic adaptation that enables breast cancer metastatic colonization. bioRxiv.

[138] Tedeschi P.M., Lin H., Gounder M. (2015). Suppression of cytosolic NADPH pool by thionicotinamide increases oxidative stress and synergizes with chemotherapy. Mol Pharmacol.

[139] De Braekeleer E., Hsu J., Hervieu C. (2018). The nucleotide kinase Nadk is required for ROS detoxification and constitutes a metabolic vulnerability of NOTCH1-driven T-ALL. Blood.

[140] Kerk S.A., Papagiannakopoulos T., Shah Y.M., Lyssiotis C.A. (2021). Metabolic networks in mutant KRAS-driven tumours: tissue specificities and the microenvironment. Nat Rev Cancer.

[141] Hsieh Y.C., Tedeschi P., Adebisi Lawal R. (2013). Enhanced degradation of dihydrofolate reductase through inhibition of NAD kinase by nicotinamide analogs. Mol Pharmacol.

[142] Nogueira V., Hay N. (2013). Molecular pathways: reactive oxygen species homeostasis in cancer cells and implications for cancer therapy. Clin Cancer Res.

[143] Zhou C.C., Yang X., Hua X. (2016). Hepatic NAD^+^ deficiency as a therapeutic target for non-alcoholic fatty liver disease in ageing. Br J Pharmacol.

[144] Sun T., Ding C.C., Zhang Y. (2022). MESH1 knockdown triggers proliferation arrest through TAZ repression. Cell Death Dis.

[145] Abshire E.T., Hughes K.L., Diao R. (2020). Differential processing and localization of human Nocturnin controls metabolism of mRNA and nicotinamide adenine dinucleotide cofactors. J Biol Chem.

[146] Luongo T.S., Eller J.M., Lu M.J. (2020). SLC25A51 is a mammalian mitochondrial NAD^+^ transporter. Nature.

[147] Kory N., Uit de Bos J., van der Rijt S. (2020). MCART1/SLC25A51 is required for mitochondrial NAD transport. Sci Adv.

[148] Girardi E., Agrimi G., Goldmann U. (2020). Epistasis-driven identification of SLC25A51 as a regulator of human mitochondrial NAD import. Nat Commun.

